# Looking After HER HEART; Let’s Talk About Women’s Heart Health

**DOI:** 10.1016/j.cjco.2023.12.019

**Published:** 2024-01-11

**Authors:** Colleen M. Norris, Sharon L. Mulvagh

**Affiliations:** a Faculty of Nursing, University of Alberta, Edmonton, Alberta, Canada; b Faculty of Medicine & School of Public Health, University of Alberta, Edmonton, Alberta, Canada; c Women and Children’s Health Research Institute, University of Alberta, Edmonton, Alberta, Canada; d Division of Cardiology, Dalhousie University, Halifax, Nova Scotia, Canada; e Department of Cardiovascular Medicine, Mayo Clinic, Rochester, Minnesota, USA

The 2023 Heart and Stroke Foundation of Canada report describes that in half the women who experience a myocardial infarction (MI), the symptoms are not recognized, with the sobering outcome that every 16 minutes a woman in Canada dies as a result of heart disease or stroke.^1^ It comes as no surprise, then, that cardiovascular disease is the leading cause of premature death in women.^1^ These statistics are attributed in part to the lack of public understanding and/or awareness of female-specific risks associated with heart health issues, but they also result from a lack of awareness on the part of clinicians who label women’s symptoms as “atypical” or “low-risk” based on risk-stratification models and evidence developed and validated primarily in men. In her book *Doing Harm; The Truth About How Bad Medicine and Lazy Science Leave Women Dismissed, Misdiagnosed and Sick*, Maya Dusenbery, editor of the award-winning Web site feministing.com, notes that the myths about heart disease being a “man’s disease” are still prevalent.^2^ She points out that when women interact with healthcare providers whose perceptions of women’s risk of heart disease continue to be influenced singularly by the male paradigm and are not viewed through a sex and gender lens, the unfortunate result is that women are less likely to be diagnosed and treated appropriately.^2^ Furthermore, the considerable body of cardiovascular research, including trials used to create clinical practice guidelines, that frames the presentation and symptoms of women with heart health issues within a predominantly male paradigm, results in women with lived experience being “stopped at the gate”^3^ of accessing care, labelled as having anxiety associated with noncardiac disorders, and turned away from further evaluation and investigations. The reasons for this situation are multifactorial; they range from unconscious or conscious bias to a lack of education and awareness. These disparities are further compounded for women of certain ethnicities, and for racialized and elderly women. In these cases, racism and ageism also enter into the intersectional dynamic of failure to access and/or receive appropriate, evidence-based care. Our own recent study reported that women who had experienced a heart health event and were attempting to navigate the healthcare system were “embarrassed” to the point of hiding the event, due to the belief that they were being stigmatized and held responsible for “not taking care of their heart—eating too much of the wrong foods or not exercising enough.”^4^

Recently, epidemiologic evidence has identified that MIs continue to increase in women aged *under* 65 years, especially in those eventually diagnosed with nonobstructive coronary arteries (MINOCA), which is more than twice as prevalent in women as it is in men.^1^ Similarly, angina, a characteristic symptom of ischemic heart disease, in women, especially younger women, is more likely to be due to ischemia associated with nonobstructive coronary arteries (INOCA).^1^ Moreover, coronary artery spasm and coronary microvascular dysfunction represent a major cause of ischemic heart disease in middle-aged women,^5^ and these entities are difficult to diagnose, requiring additional specialized testing with limited availability. As well, spontaneous coronary artery dissection (SCAD), a common cause of MIs in younger women—with 90% of SCAD patients being women^6^^,^^7^—was previously thought to be rare, until large registries in North America, and now globally, have demonstrated otherwise. SCAD now accounts for 25% to 30% of all MIs in women aged under 60 years, and over 40% of MIs in women aged less than 40 years,^6^^,^^7^ and it is the most common cause of MIs in pregnancy and the peripartum period.^8^ From a life-stage perspective, the influences of sex hormones on the regulation of biological and physiological processes have not translated into the development, collection, or analyses of data on treatments and outcomes in cardiovascular disease (CVD) management in women. As a result, the gap in our understanding of the mechanistic relationship between the sex (biological) and gender (psychosociocultural) factors in CVD means that women with heart health issues continue to be underresearched, underdiagnosed, undertreated, undersupported, and under-aware.^1^^,^^9^

Henry Ford once said that “If everyone is moving forward together, then success takes care of itself,”^10^ and this *CJC Open* special issue, with a focus on women’s heart health, clearly demonstrates that we are truly moving forward in using the evidence available to inform practice, as well as using practice to demonstrate the evidence we yet need to inform and treat women’s health.

## Evidence to Inform Practice

In 2018, the Canadian Women’s Heart Health Alliance (CWHHA; Web site: https://www.cwhha.ca) was established as a network of experts and advocates to develop and disseminate evidence-informed strategies to transform clinical practice and enhance collaborative action on women’s cardiovascular health in Canada. The “superpower” of the CWHHA is that the membership is inclusive, consisting now of over 200 members from across Canada and including clinicians, scientists, allied health professionals, program administrators, and patient partners, with the goal of improving women’s cardiovascular health across the lifespan. The mission “to support clinicians, scientists, patients and decision-makers in working collaboratively to implement evidence, transform clinical practice and impact public policy related to women’s cardiovascular health” was actioned by establishing 4 working groups addressing the following: advocacy; training and education; knowledge translation and mobilization; and health systems and policy. Early on, following the establishment of the CWHHA and the development of working group projects, our working groups on health systems and policy and knowledge translation and mobilization (which we, C.M.N. and S.L.M., respectively, led), perceived the value in collaboration, and sought and received committed support from Dr Michelle Graham (editor of *CJC Open*) to co-create *The CWHHA ATLAS on the Epidemiology, Diagnosis, and Management of Cardiovascular Diseases in Women*, establishing a special CWHHA Collection tab (https://www.cjcopen.ca/womens_heart_health_alliance).^11^ The *CWHHA ATLAS* project had its roots in the seminal “State of the Science in Women's Cardiovascular Disease: A Canadian Perspective on the Influence of Sex and Gender” publication in the “Go Red” issue of the *Journal of the American Heart Association* in 2020.^11^,^12^ The *CWHHA ATLAS* has focused on presenting “deep dives” into the “understudied, underdiagnosed, and undertreated” aspects of women’s heart health, creating multi-chapter, in-depth, contemporary reviews of the current evidence, including the epidemiology, diagnosis, and management of CVD to inform practice for women across the lifespan.

It is fitting, then, that the final 2 chapters of the *CWHHA* “Chapter 8: Knowledge Gaps and Status of Existing Research Programs in Canada”^13^ and “Chapter 9: Summary of Current Status, Challenges, Opportunities, and Recommendations”^14^—are published in this special *CJC Open* #HerHeartMatters issue during Heart Month in Canada. These chapters highlight the programs of research being undertaken across Canada and summarize opportunities and recommendations for moving women’s cardiovascular health forward. In addition, this *CJC Open* special issue includes a number of articles that provide state-of-the-science data in areas in CVD diagnosis, and treatments specific to disproportionately female–predominant conditions. Examples are the following: “What Is New in Spontaneous Coronary Artery Dissection?;”^15^ “Pathophysiology of Myocardial Infarction With Nonobstructive Coronary Artery Disease: A Contemporary Systematic Review;”^16^ “Antithrombotic Management and Outcomes of Anterior ST-Elevation Myocardial Infarction With New-Onset Wall Motion Abnormalities in Men and Women;”^17^ and “Female-Specific Considerations in Aortic Health and Disease.”^18^ Sex- and gender-unique aspects of cardiovascular risk across the lifespan of a woman are also addressed, including the following: “Prevalence of Sex-Specific Cardiovascular Disease Risk Factors, Medical Risk, and Engagement in Health-Promoting Behaviours in Premenopausal Females;”^19^ “The Importance of Nontraditional and Sex-Specific Risk Factors in Young Women With Vasomotor Nonobstructive vs Obstructive Coronary Syndromes;”^20^ “Increased Prevalence of Adverse Health Outcomes Across the Lifespan in Those Affected by Polycystic Ovary Syndrome: A Canadian Population Cohort;”^21^ and “Women’s Heart Health and the Menopausal Transition: Two Faces of the Same Coin.”^22^ Unique aspects of hypertension in women with renal dysfunction as a CVD risk factor are also explored in “Awareness of Hypertension in Reproductive-Aged Women Living With Chronic Kidney Disease”^23^ and “The Association Between Testosterone and Vascular Function in Reproductive-Aged Females With Chronic Kidney Disease.”^24^

## Practice-Based Evidence

We were also fortunate to be able to include articles that address existing clinical practice models of CVD care for women in the context of their perspectives and preferences regarding care delivery, to identify and contribute to development of a successful foundation for clinical practice standards and guidelines These articles include a spectrum of local, regional, national, and global reports—again, across a woman’s lifespan: “Understanding Patient Perspectives on Specialized, Longitudinal, Postpartum, Cardiovascular Risk-Reduction Clinics;”^25^ “Nutrition Interventions for Lowering Cardiovascular Risk After Hypertensive Disorders of Pregnancy: A Systematic Review;”^26^ and “Women-Focused Cardiac Rehabilitation Delivery Around the World and Program Enablers to Support Broader Implementation.”^27^ Finally, we conclude on an optimistic note, with a report on “Sex, Gender, and Women’s Heart Health: How Women’s Heart Programs Address the Knowledge Gap.”^28^

In essence, this special #HerHeartMatters Heart Month issue on women’s heart health is celebrating all those who have continued to move the dial on advancing the awareness and evidence regarding women’s heart health, not only in Canada but also internationally.^29^ More importantly, it is a “call to action,” challenging us all to continue the work in identifying, diagnosing, and treating the sex- and gender-specific aspects of women’s heart health. We must continue to build the evidence base, and strive toward guideline-directed sex- and gender-focused diagnoses and treatments, to improve cardiovascular outcomes for all women.
